# Antimicrobial effects and mechanism of action of carboxymethyl chitosan-loaded silver ion complexes against drug-resistant *Aspergillus fumigatus*

**DOI:** 10.71150/jm.2512001

**Published:** 2026-04-06

**Authors:** Lingsheng Jin, Xinyu Zhou, Wenlong Du

**Affiliations:** 1 Department of Bioinformatics, School of Life Sciences, Xuzhou Medical University, Xuzhou, Jiangsu 221004, P. R. China; 2 Department of Biophysics, School of Life Sciences, Xuzhou Medical University, Xuzhou, Jiangsu 221004, P. R. China

**Keywords:** drug-resistant *Aspergillus fumigatus*, carboxymethyl chitosan, silver ions, antimicrobial, biofilm

## Abstract

Based on the escalating challenge of drug-resistant *Aspergillus fumigatus* infections, this study developed a silver ion-loaded carboxymethyl chitosan (CMCh-Ag^⁺^) nanocomposite as a potent antifungal agent. The composite was successfully synthesized and characterized, revealing distinct physicochemical properties, uniform dispersion, and confirmed coordination between CMCh and Ag^⁺^. In vitro evaluations, including minimum inhibitory concentration (MIC), minimum fungicidal concentration (MFC), growth curve, and plate spotting assays, demonstrated that CMCh-Ag^⁺^ exhibited significantly superior antifungal efficacy against multiple *A. fumigatus* strains (including azole-resistant isolates) compared to CMCh or Ag^⁺^ alone. In vivo experiments using a *Galleria mellonella* infection model confirmed the enhanced therapeutic effect and biocompatibility of CMCh-Ag^⁺^. Investigations into the mechanism-related phenotypes revealed that CMCh-Ag^⁺^ significantly removed fungal biofilm and was associated with a substantial accumulation of intracellular reactive oxygen species (ROS), correlating with fungal cell death. This research highlights the preliminary potential of CMCh Ag^⁺^ as a candidate strategy to combat drug-resistant *A. fumigatus* infections, warranting further investigation in mammalian models to assess its clinical translational prospects.

## Introduction

*A. fumigatus*, a ubiquitous filamentous fungus found in natural environments, is a clinically relevant opportunistic pathogen that can colonize the human respiratory system and cause pulmonary aspergillosis (Abad et al., 2010). This species presents substantial risks to public health (Lin et al., 2001), and the increasing prevalence of drug-resistant *A. fumigatus* strains has further complicated the therapeutic management of infections in clinical settings (De Francesco, 2023).

Invasive aspergillosis (IA) (Latgé and Chamilos, 2019), which is caused primarily by *A. fumigatus*, represents a significant threat to immunocompromised populations, including organ transplant recipients, individuals living with HIV/AIDS, patients receiving chemotherapy or radiotherapy for malignancies, and those receiving long-term immunosuppressive therapies (Douglas et al., 2021). Epidemiological studies indicate that the incidence of IA in high-risk groups ranges from 10% to 25%, with mortality rates increasing dramatically to between 40% and 90%. The emergence of drug-resistant strains of *A. fumigatus* has further intensified this crisis, severely undermining the effectiveness of conventional antifungal treatments—such as amphotericin B (Fakhim et al., 2022), fluconazole (van der Linden et al., 2015), and voriconazole (Latgé, 1999)—in clinical practice. Mechanistically, these resistant isolates evade therapeutic action through various strategies (Zargaran et al., 2017), including modifications at target sites and increased efflux pump activity. These adaptations ultimately lead to treatment failure and life-threatening complications (Zhang and Cheng, 2022).

Confronting the pressing challenge posed by drug-resistant *A. fumigatus*, the development of novel antifungal agents has attained unprecedented urgency. Recent advancements underscore the significant potential of both metal ions and biopolymeric materials for antimicrobial applications (Mohamed et al., 2019) where strategic integration promises synergistic effects with supra-additive efficacy. Specifically, silver ions (Ag^⁺^) exhibit broad-spectrum antimicrobial activity through complexation with microbial biomacromolecules (e.g., proteins, nucleic acids), thereby disrupting essential metabolic and physiological functions (Yin et al., 2020). However, the standalone application of Ag^⁺^ is fundamentally limited by poor colloidal stability, a propensity for aggregation, immunotoxicity that is dose dependent (Li et al., 2020; Orta-García et al., 2015), and environmental persistence, the latter contributing to ecotoxicological risks in aquatic systems (McGillicuddy et al., 2017), which severely constrains its clinical utility.

Carboxymethyl chitosan (CMCh), a notable derivative of chitosan, is synthesized through chemical modification that introduces carboxymethyl groups onto the chitosan backbone (Dong et al., 2010). This engineered biomaterial retains the inherent advantages of chitosan—including excellent biocompatibility, biodegradability, and intrinsic antimicrobial activity—while exhibiting enhanced aqueous solubility and superior ion-exchange capacity due to its carboxymethyl functionalities (Shariatinia, 2018). Importantly, the presence of reactive groups (e.g., carboxyl and amino moieties) within CMCh molecules facilitates efficient metal ion chelation, thereby establishing its utility as an exceptional scaffold for coordinating metal ions (Huang et al., 2019).

The integration of CMCh with silver ions results in the formation of CMCh-Ag^⁺^ nanocomposites, which effectively address the inherent limitations of both components while leveraging their synergistic antimicrobial properties (Yan et al., 2020). As a molecular scaffold, CMCh efficiently disperses and stabilizes Ag^⁺^ ions to prevent aggregation, thus enhancing antimicrobial efficacy and colloidal stability (Fan et al., 2022; Huang et al., 2023). This complex exhibits multifaceted antifungal effects against drug-resistant *A. fumigatus* through complementary mechanisms: the CMCh component binds to anionic groups on the fungal cell membrane, compromising structural integrity and increasing permeability (Ma et al., 2021), which facilitates the influx of Ag^⁺^ into the cytoplasm, where it complexes with intracellular biomacromolecules (e.g., proteins and DNA), disrupting essential metabolic pathways (Buglak and Nguyen, 2024). Concurrently, the intrinsic immunomodulatory activity of CMCh (Wu et al., 2024) enhances host defense mechanisms, allowing for reduced dosing of Ag^⁺^ to minimize potential systemic toxicity while maintaining therapeutic effectiveness.

This study employs traditional methods to synthesize CMCh using chitosan as the raw material. The successful synthesis of CMCh loaded with silver ions was confirmed through ultraviolet and infrared absorption spectroscopy. Scanning electron microscopy was used to observe the three-dimensional structure and dispersion degree of the material. We subsequently conducted comparative assessments of antimicrobial efficacy through minimum inhibitory concentration (MIC), minimum fungicidal concentration (MFC), plate counting experiments, and cell growth curve analyses for both single agents and composite agents. In vivo experiments were performed on *Galleria mellonella* to evaluate the biocompatibility of the agents and its actual antimicrobial effects within a biological system. Finally, ROS measurement assays and cell membrane clearance experiments were carried out to investigate the antimicrobial mechanisms involved. This research aims to provide new insights into drug-resistant *A. fumigatus* infections, demonstrating significant potential applications in clinical settings.

## Materials and Methods

### Reagents

Silver nitrate and chitosan were obtained from Sinopharm Group Chemical Reagent Co., Ltd. (China). Sodium hydroxide and ammonium citrate were obtained from Shanghai Macklin Biochemical Co., Ltd. (China), which also supplied resazurin. Anhydrous methanol was sourced from Beijing InnoKai Technology Co., Ltd. The 1× PBS buffer solution was purchased from Yida Technology (Quanzhou) Co., Ltd. Chloracetic acid was acquired from Shanghai Maikelin Biochemical Technology Co., Ltd., whereas dichlorofluorescein diacetate was obtained from Shanghai Biosharp Biotechnology Co., Ltd. Glacial acetic acid was sourced again from Shanghai Maikelin Biochemical Technology Co., Ltd.

### Preparation of strains

This study investigated a total of four strains, specifically *A. fumigatus* 1161 (wild type), ED-22G, ED-23G (isolated from the environment), and Shjt 40 and Shjt 42b (isolated from clinical patients) (Chen et al., 2024b). The latter four strains demonstrated resistance to azole antifungals (Du et al., 2021, 2023). *A. fumigatus* was preserved at -20°C in a solution of 50% glycerol. Unless otherwise specified, the concentration of fungal suspensions used in subsequent experiments was maintained at 1 × 10^6^ CFU/ml.

### Preparation of carboxymethyl chitosan

Using an analytical balance, 0.5 g of chitosan was accurately weighed and dissolved in 10 ml of a 20% (w/v) NaOH solution. Following this, 1.5 g of chloroacetic acid was added under continuous magnetic stirring. The reaction mixture was maintained at a temperature of 40°C for a duration of 2 h. After the reaction, the mixture was neutralized to pH 7.0 using a 10% (v/v) glacial acetic acid solution. The neutralized mixture was then centrifuged at 12,000 rpm for 15 min, and the precipitate was collected. This precipitate was dissolved in a suitable amount of methanol and subjected to another centrifugation at 12,000 rpm in a high-speed centrifuge for 15 min. The final precipitate was collected. The precipitate was placed in a freeze dryer with the critical parameters set as follows: a cold trap temperature of -72°C and a vacuum level of 12 Pa. Under these conditions, it was freeze-dried for 8 h to obtain carboxymethyl chitosan powder. The resulting powder was sealed and stored in a dark, dry place for future use.

The CMCh-Ag^⁺^ complex was prepared using a chemical synthesis method with carboxymethyl chitosan (CMCh) acting as both reducing agent and stabilizer. Specifically, 0.1024 g of silver nitrate (AgNO_3_) and 0.1024 g of CMCh were dissolved in 100.0 ml of sterile ultrapure water. The mixture was subjected to continuous magnetic stirring at room temperature for 6 h, resulting in a CMCh-Ag^⁺^ solution with a concentration of 2048 μg/ml. This solution was stored in a refrigerator at 4°C for later use. An appropriate amount of the above CMCh-Ag^⁺^ solution was transferred to a freeze dryer. The critical freeze-drying parameters were confirmed as follows: cold trap temperature of -72°C and vacuum level of 12 Pa. Freeze-drying was carried out for 8 h to remove water and obtain the solid CMCh-Ag^⁺^ complex powder. The freeze-dried powder was stored in a refrigerator at 4°C for subsequent research use (Wahid et al., 2017).

### Refinement of complex characterization

An appropriate volume of sample solution was used for the analysis of particle size and distribution. A suitable amount of sample solution was transferred into a cuvette, and distilled water was used as a blank reference. The ultraviolet absorption spectrum was measured within the wavelength range of 200–800 nm. The sample mixture was subjected to rotary evaporation at 60°C to obtain a solid powder. This powder was subsequently placed in a vacuum drying oven at 50°C for 2 h. The resulting powdered sample was then used for Fourier transform infrared (FTIR) characterization. An appropriate volume of sample solution was collected for scanning electron microscopy analysis.

### Research on the antimicrobial properties of complexes

**Minium inhibitory concentration (MIC) test:** The five strains of *A. fumigatus* were diluted in MM liquid culture medium to a concentration of 1 × 10^6^ CFU/ml. A series of different concentrations of the three types of agents were prepared via a twofold dilution method across 21 sterile centrifuge tubes. The specific dilution protocol is detailed in the table below. In each well of a sterile 96-well plate, 100 μl of the diluted agent solution and 100 μl of the fungal suspension were added, followed by incubation at 37°C for 36 h. After the liquid from each well was removed, an additional 100 μl of culture medium containing 0.002% methylene blue was added to each well, and the mixture was incubated at 37°C for another 4 h. The color change was then observed; the first well that exhibited a blue color indicated the corresponding agent concentration as the MIC (Espinel-Ingroff et al., 2011).

**Minimum fungicidal concentration (MFC) test:** *A. fumigatus* conidial suspensions (five strains) were prepared and adjusted to a concentration of 1 × 10^6^ CFU/ml in MM according to CLSI guidelines. The test compounds were serially diluted twofold in MM, ranging from 512 to 16 μg/ml. In sterile 96-well plates, 100 μl of each agent dilution was combined with 100 μl of the conidial suspension and incubated at 37°C for 24 h. Subsequently, aliquots (200 μl) from each well were plated onto MM agar and streaked three times via sterile swabs, ensuring a rotation of 60° between streaks, followed by perimeter streaking. After the plates were allowed to dry at room temperature for five min, they were inverted and incubated at 37°C for an additional 36 h. The MFC was defined as the lowest agent concentration that resulted in ≤ 0.1% survival relative to the initial inoculum (Manavathu et al., 2000).

**Microbial growth curve:** The five strains of *A. fumigatus* were diluted in MM medium to a concentration of 1 × 10^6^ CFU/ml. The compounds were then diluted to the corresponding concentrations of the MIC, 1/2 MIC, and 1/4 MIC. In a sterile 96-well plate, 100 μl of the prepared agent solutions at different concentrations was added to each well, followed by the addition of 100 μl of the five strains of *A. fumigatus* suspension. The plates were incubated in a constant-temperature incubator at 37°C. Every two h, the optical density (OD) values were measured at a wavelength of 600 nm via an enzyme-labelled instrument for a total duration of 36 h (Furukawa et al., 2020).

**Plate spotting experiment:** On the surface of the prepared MM agar plates containing different concentrations of agents, 2 μl each of the five *A. fumigatus* suspensions were slowly added at concentrations of 1 × 10^8^ CFU/ml, 1 × 10^7^ CFU/ml, and 1 × 10^6^ CFU/ml. The inoculated MM agar plates were subsequently placed in a constant-temperature incubator at 37°C for incubation for 24 h. The growth status of *A. fumigatus* colonies on the surface of the culture medium was observed.

**In vivo antimicrobial study of the compound:** The spore suspension of *A. fumigatus* (Shjt 40) was diluted in MM liquid culture medium to achieve a final concentration of 1 × 10^6^ CFU/ml. Both CMCh-Ag^+^ and Ag^+^ solutions were diluted to a final concentration of 32 μg/ml. A total of 60 healthy *G. mellonella* larvae were selected and randomly divided into six groups labelled a through f, with each group containing 10 individuals. Group a served as the control group and received an injection of 20 μl of physiological saline into the second right leg via a microinjector. Groups b and c underwent single agent treatment and received injections of either the diluted Ag^+^ or CMCh-Ag^+^ solution at a volume of 20 μl each. Group d was inoculated with *A. fumigatus* by injecting 20 μl of the diluted spore suspension. Groups e and f represented the combination treatment groups; they first received an injection of 10 μl of spore suspension followed by an injection of either Ag^+^ or CMCh-Ag^+^ solution at a volume of 10 μl one h later. Larval mortality was observed every 24 h, and mortality curves were plotted accordingly (Sun et al., 2022).

### Research on the antimicrobial mechanisms of complexes

**Biofilm removal experiments:** One hundred microlitres of the diluted fungal suspension was added to a sterile 96-well plate and incubated at 37°C for 24 h to allow biofilm formation. After incubation, the fungal mixture was aspirated and washed with PBS to remove any adherent pathogenic fungi. Agent combinations at various concentrations were prepared, after which 100 μl of each combination was added to separate sterile wells in the same plate. The medium group served as a blank control. The culture medium from each well was aspirated and fixed with 100 μl of methanol for 15 min. The wells were stained with crystal violet for an additional period of ten min. Finally, the wells were washed three times with PBS. Any residual biofilm was dissolved in absolute ethanol (95%), and OD was measured at a wavelength of 600 nm via a microplate reader (Pierce et al., 2008). The biofilm eradication concentration (BEC_50_) was defined as the minimum concentration causing 50% reduction in biofilm biomass, determined by crystal violet staining (OD_600_).

**Determination of reactive oxygen species (ROS):** The ED-23G *A. fumigatus* solution was diluted in MM liquid culture medium to achieve a working concentration of 1 × 10^6^ CFU/ml. A sterile black 96-well plate was prepared, and 100 μl of the diluted ED-23G was inoculated into each well. The plate was then placed in a constant-temperature incubator at 37°C for 12 h to promote mycelial formation. The three agents were diluted with sterile PBS to a final concentration of 32 μg/ml for use. After an incubation period of 12 h, the culture medium within each well was removed, and the mycelia were washed twice with sterile PBS solution. The diluted agents were subsequently added to their respective wells and incubated at 37°C for an additional 3 h. Upon completion of treatment, excess agent solutions were aspirated from the wells, followed by two washes with PBS. Under light-protected conditions, 2′,7′-dichlorodihydrofluorescein diacetate (H₂DCFH‑DA) dye was added to achieve a final concentration of 40 μg/ml, and the mixture was incubated at 37°C in darkness for one h. The stained mycelia were analysed under an excitation wavelength of 488 nm; the fluorescence emission intensity at a wavelength of 525 nm was recorded to assess changes in the intracellular ROS levels via a multifunctional microplate reader for data analysis (Jambunathan, 2010).

## Results

### Characterization of complexes

**Fig. 1.**
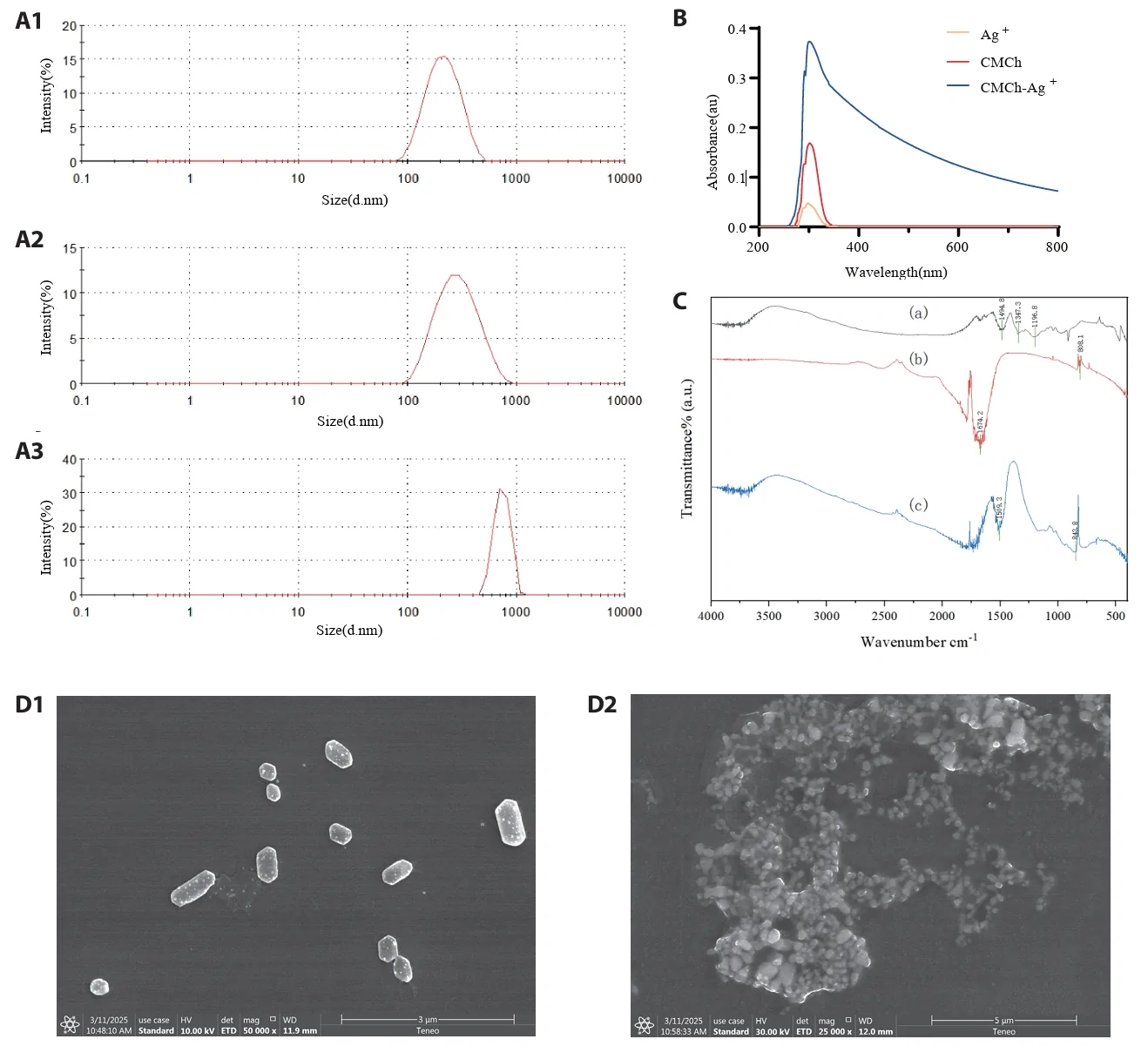
Characterization of CMCh-Ag^⁺^ complexes. (A) Size distribution of the composite materials; (A1) Size distribution of Ag^⁺^; (A2) Size distribution of CMCh; (A3) Size distribution of CMCh-Ag^⁺^; (B) Ultraviolet absorption spectrum of the complex within the range of 200–800 nm; (C) Fourier transform infrared (FTIR) absorption spectra of CMCh (a), Ag^⁺^ (b), and CMCh-Ag^⁺^ (c). (D) Scanning electron microscopy images of CMCh (D1) and CMCh-Ag^⁺^ (D2).

To investigate the changes in the morphology and size distribution of CMCh before and after the formation of CMCh-Ag^+^ complexes, we characterized the dried samples of the three agents using scanning electron microscopy (SEM). As shown in Fig. 1A, the Ag^+^ sample exhibited aggregated structures with sizes ranging from approximately 80 to 600 nm; the CMCh sample showed block-like structures varying between 90 and 1,000 nm; and the CMCh-Ag^+^ composite formed loose aggregates with sizes between 400 and 1,100 nm. The polydispersity indices (P.I.) were measured as follows: 0.773 for Ag^+^, 0.830 for CMCh, and 0.461 for CMCh-Ag^+^, indicating that the CMCh-Ag^+^ composite displayed a relatively more uniform aggregation state.

To investigate the changes in functional groups and chemical bonds before and after the formation of the complexes, ultraviolet absorption spectroscopy experiments were conducted on three substances. As shown in Fig. 1B, the results indicate that a characteristic peak appears at approximately 301 nm in the UV absorption spectrum of the CMCh-Ag^+^ complex. This peak is attributed to the excitation of surface plasmon resonance (SPR) associated with Ag^+^ ions. The presence of this characteristic SPR band for CMCh-Ag^+^ suggests that functional groups such as amino and carboxyl groups in CMCh interact with Ag^+^ ions. It can be concluded that a coordination bond is formed between them, which alters the electronic cloud distribution and conjugated system within the molecules. Consequently, this change affects both their light absorption capacity and absorption wavelength, leading to variations in both peak position and intensity. In contrast, no absorption peaks were observed in the chitosan solution because of the absence of Ag^+^ ions.

To further analyse the structure of the complex and the interactions within it, infrared absorption spectroscopy measurements were conducted before and after the formation of the complex. As shown in Fig. 1C, in the FTIR spectrum of CMCh, the peak at approximately 1600–1650 cm^-1^ is associated with the amide I band (C=O stretching vibration), whereas the peak at approximately 1500–1550 cm^-1^ corresponds to the amide II band (N–H bending vibration and C–N stretching vibration). AgNO_3_ exhibits a strong absorption peak at approximately 1480–1720 cm^-1^, which is related primarily to NO^3-^ and corresponds to its asymmetric stretching vibration; an additional absorption peak is attributed to NO^3-^'s in-plane bending vibrations at approximately 830–870 cm^-1^. These characteristic peaks allow for the identification of AgNO_3_. After CMCh forms a complex with Ag^+^ (CMCh-Ag^+^), corresponding changes occur in its infrared absorption spectrum. The coordination interaction between Ag^+^ and certain functional groups in CMCh (such as -OH, -NH_2_, and -COO^-^) results in shifts and broadening of the stretching vibration peaks for -OH and -NH_2_; similarly, both the asymmetric and symmetric stretching vibration peaks for -COO^-^ are altered due to this coordination effect. This led us to conclude that there was an interaction between Ag^+^ and CMCh, confirming the formation of CMCh-Ag^+^.

To observe the specific structural characteristics of the complexes more intuitively, scanning electron microscopy (SEM) experiments were conducted on CMCh-Ag^+^ and CMCh. SEM uses raster scanning technology to obtain magnified images by focusing an electron beam onto the sample surface, which scans and generates energy loss that excites secondary electrons and other signals. These signals are then detected and converted into images. Owing to its large depth of field, SEM can clearly present the three-dimensional morphology of samples. As shown in Fig. 1D, CMCh has a relatively regular block structure with pores. This structure may be related to its intrinsic intermolecular forces and crystallinity; its relatively dense block configuration has a certain effect on physical properties such as strength. In contrast, CMCh-Ag^+^ displays a loose aggregation of particles. This dispersed structure likely arises from the interaction between silver ions and chitosan during their combination process, where numerous small particles cluster together, thereby increasing the specific surface area, which is beneficial for enhancing antimicrobial activity, among other activities. The transparent coating with patterns observed on the surface corresponds to CMCh, while the particles represent encapsulated Ag^+^.

### Research on antimicrobial performance

**Fig. 2.**
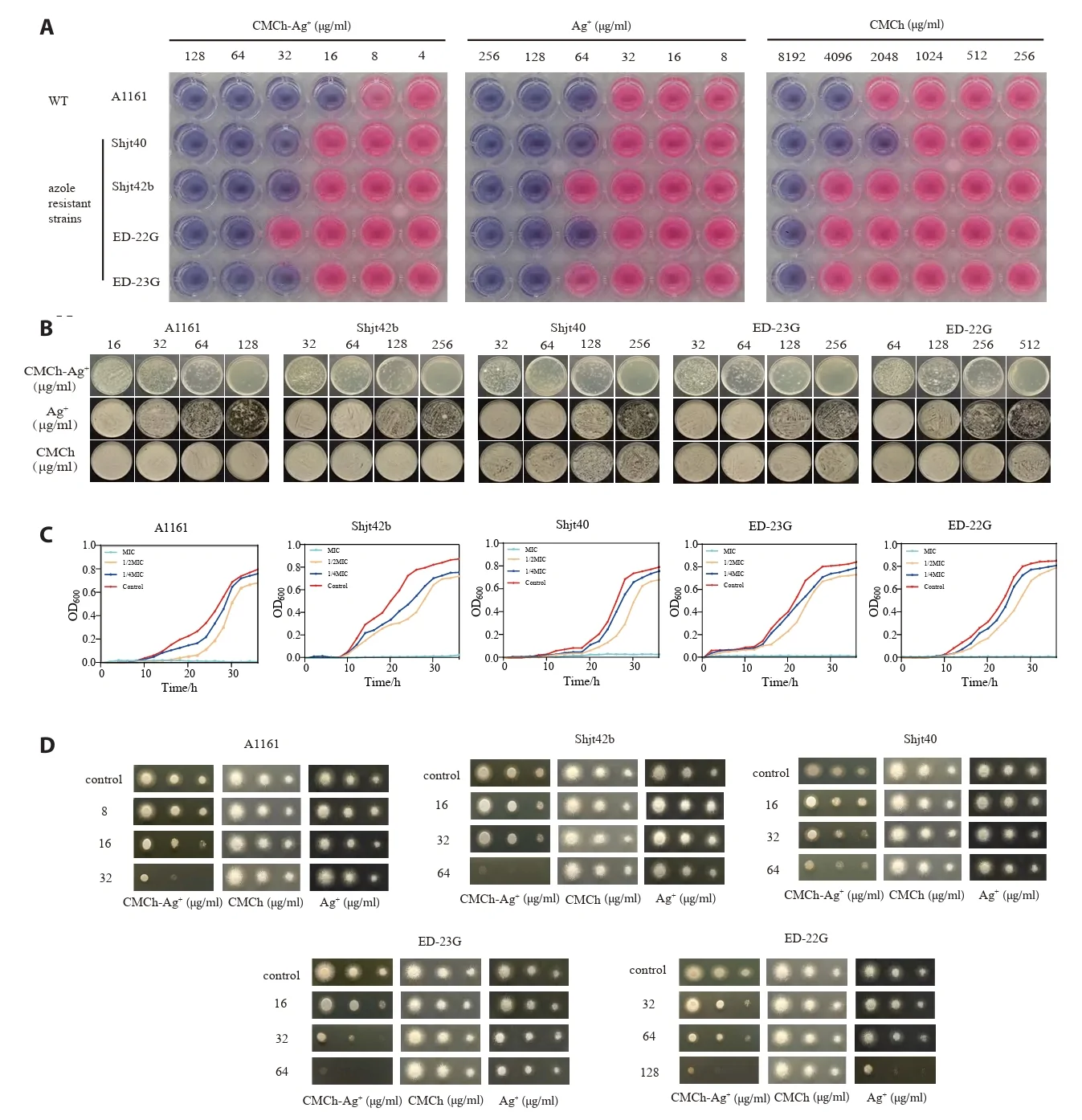
Research on the in vitro antimicrobial properties of the agents. (A) MICs of CMCh-Ag^⁺^, Ag^⁺^, and CMCh against five strains of *A. fumigatus*. In each group of pictures, medium blue represents no metabolic activity, and pink purple represents high metabolic activity. (B) MFCs of CMCh-Ag^⁺^, Ag^⁺^, and CMCh against five strains of *A. fumigatus*. (C) Fungal growth curves were generated by measuring OD_600_ via a microplate reader for different concentrations of CMCh-Ag^⁺^ in *A. fumigatus* liquid cultures over a period of 36 h, with measurements taken every 2 h. (D) Plate culture experiment. In sterilized MM agar medium, five strains of *A. fumigatus* suspensions (1 × 10^8^ CFU/ml, 1 × 10^7^ CFU/ml, and 1 × 10^6^ CFU/ml) were added to a volume of 2 μl each. The cultures were incubated for 24 h.

**In vitro antimicrobial experiments:** To investigate the antimicrobial effect of CMCh-Ag^+^ against *A. fumigatus*, a MIC experiment was conducted. As shown in Fig. 2A, the results of methylene blue staining indicate that a blue color in the culture medium represents low metabolic activity of the microbial cells, suggesting that there is an antimicrobial effect at this concentration. Conversely, if a pink‒purple color is observed, it indicates high metabolic activity and a greater number of microbial cells, implying that there is no significant antimicrobial effect at this concentration. Therefore, CMCh-Ag^+^ clearly has a more pronounced antimicrobial effect than both CMCh and Ag^+^.

As shown in Fig. 2B, the A1161 strain produced fewer than 20 colonies when cultured on plates coated with 128 μg/ml CMCh-Ag^+^. The Shjt40 and Shjt42b strains also produced fewer than 20 colonies on plates coated with 512 μg/ml CMCh-Ag^+^, whereas the ED-23G strain produced fewer than 20 colonies at a concentration of 256 μg/ml. In contrast, both CMCh and Ag^+^ resulted in colony counts significantly greater than 20 under equivalent conditions. These results indicate that neither CMCh nor Ag^+^ demonstrated effective bactericidal activity at comparable concentrations; conversely, the bactericidal efficacy of CMCh-Ag^+^ is markedly superior to that of either component alone (Table 1).

To quantitatively and visually observe the antimicrobial effects of CMCh-Ag^+^ on *A. fumigatus*, a growth curve experiment was conducted. The results presented in Fig. 2C indicate that under the conditions of MIC, 1/2 MIC, 1/4 MIC, and control concentrations of CMCh-Ag^+^ solution, the absorbance at a wavelength of 600 nm was measured over a period of 36 h for five different strains. A higher absorbance value suggests greater biomass accumulation in *A. fumigatus*. Within the 36-h timeframe, the OD_600_ value for the CMCh-Ag^+^ solution at the MIC remained consistently low, indicating no growth of *A. fumigatus*; however, the OD_600_ values in the other groups were significantly greater than those at the MIC, indicating weaker inhibitory effects and resulting in an S-shaped growth curve for *A. fumigatus*. This evidence confirms that CMCh-Ag^+^ has inhibitory effects on the growth of *A. fumigatus*.

To observe the growth of *A. fumigatus* colonies intuitively and evaluate the antimicrobial effects of three different agents, we conducted plate inoculation experiments using various strains of *A. fumigatus* on agar media containing three different agent concentrations. The results presented in Fig. 2D indicate the growth conditions after 24 h for 2 μl of different concentrations of bacterial suspensions on agar media with identical agent concentrations. Among these media, CMCh-Ag^⁺^-treated agar medium significantly inhibited the growth of the inoculated strains, resulting in smaller colony diameters and lighter colors. In contrast, both CMCh- and Ag^⁺^-treated agar media had a weaker impact on strain growth, leading to larger colony diameters and darker colors. It can be concluded that CMCh-Ag^⁺^ is more effective at inhibiting *A. fumigatus* growth than CMCh or Ag^⁺^ alone. Furthermore, there are differences in sensitivity among various strains of *A. fumigatus* and resistant variants towards CMCh, Ag^⁺^, and CMCh-Ag^⁺^; this may be attributed to their inherent physiological characteristics and resistance mechanisms.

**Fig. 3.**
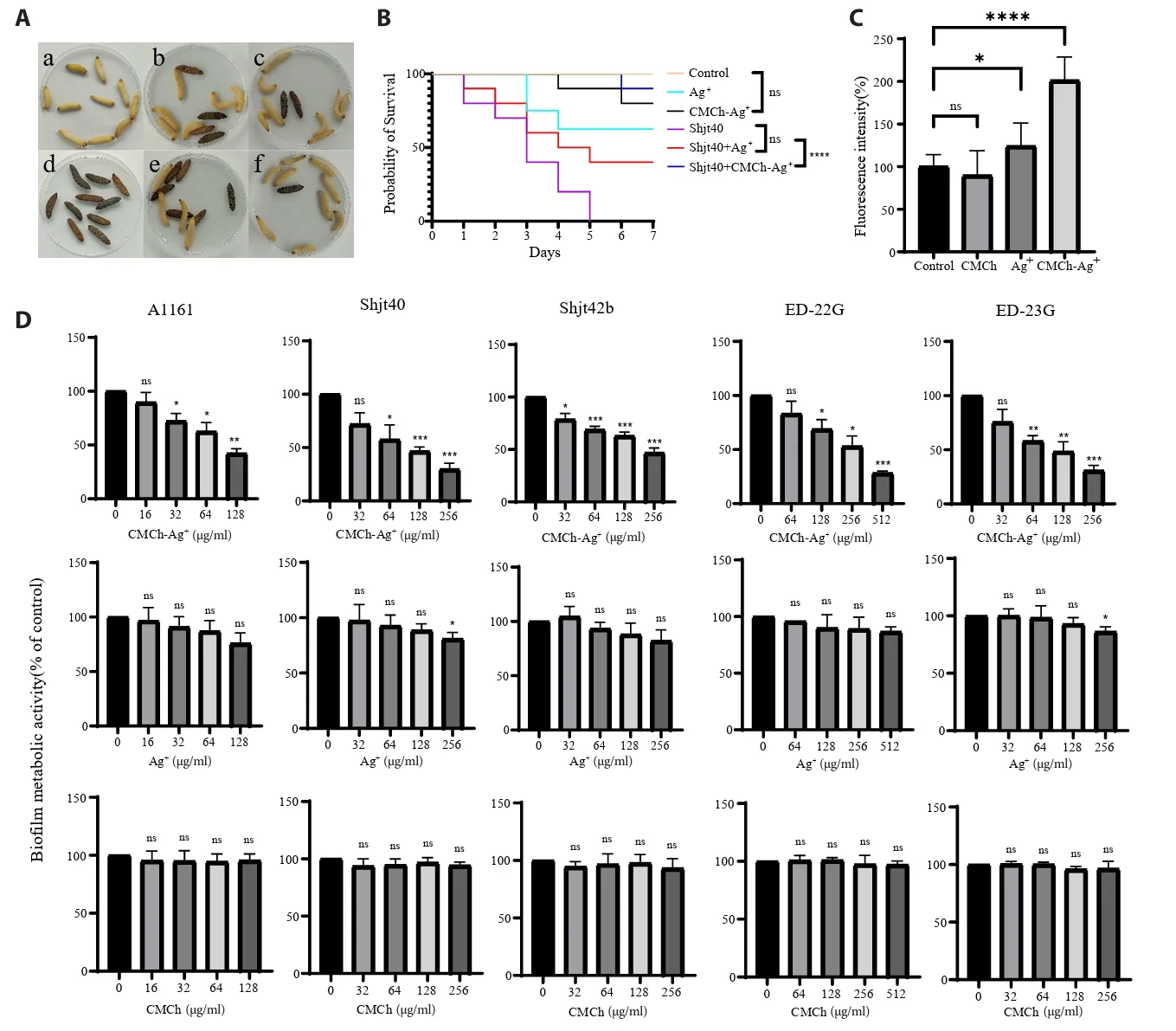
Antifungal and biofilm-removing functions of CMCh-Ag^⁺^ composite validated in *G. mellonella* and drug-resistant *A. fumigatus*. (A) antimicrobial experiment on *G. mellonella* larvae. (a) Physiological saline group; (b) Ag^⁺^ treatment group; (c) CMCh-Ag^⁺^ agent treatment group; (d) ED-23G fungal treatment group; (e) ED-23G mixed with Ag^⁺^ treatment group; (f) ED-23G mixed with CMCh-Ag^⁺^ treatment group. Dead individuals are dark brown, and surviving individuals are white. (B) Survival curve of *G. mellonella* larvae. ****, *p* < 0.0001; ns, *p* > 0.05 according to log rank analysis. (C) Effects of CMCh, Ag^⁺^, and CMCh-Ag^⁺^ on intracellular ROS levels in drug-resistant *A. fumigatus* (strain ED-23G). Fluorescence intensity reflects intracellular ROS concentration. ns, *p* > 0.05; **, *p* < 0.01; ****, *p* < 0.0001 according to log rank analysis. (D) Valuation of the biofilm clearance efficacy of three different agents against five strains of *A. fumigatus* at various concentrations. OD_600_ was measured via a microplate reader following crystal violet staining. *, *p* < 0.1; **, *p* < 0.01; ***, *p* < 0.001; ns, *p* > 0.05 according to log rank analysis.

**In vivo antimicrobial experiments:** To investigate the antimicrobial effects of three different agents within a biological system, an in vivo experiment was conducted using *G. mellonella* larvae. As shown in Fig. 3A and Fig. 3B, both the Ag^⁺^ and CMCh-Ag^⁺^ treatment groups exhibited larval mortality, indicating that these two treatments cause agent toxicity. The survival curves revealed no significant differences between the two groups receiving agent injections and the saline control group, suggesting that the agents themselves do not exhibit notable toxicity toward *G. mellonella* larvae. Among the three groups subjected to bacterial inoculation, both agent treatment groups presented lower mortality rates than did the group injected solely with bacterial mixture, indicating that these two agents have certain antimicrobial effects in vivo. Notably, the CMCh-Ag^⁺^ group had the lowest number of deaths, indicating its most pronounced antimicrobial efficacy.

### Research on antimicrobial mechanism-related phenotypes

To investigate the effect of these three types of agents on *A. fumigatus* biofilms, crystal violet staining assays were conducted to assess changes in total biofilm biomass after agent treatment at different concentrations across various strains. As shown in Fig. 3D, treatment with CMCh-Ag^+^ resulted in a significant reduction in crystal violet staining intensity compared to the control, indicating a decrease in total biofilm biomass or biofilm-associated cell viability. In contrast, CMCh or Ag^+^ alone showed minimal effects under the same conditions. The effectiveness of biofilm clearance improved with increasing concentrations of CMCh-Ag^⁺^. The BEC50 value for strains A1161, Shjt40, and ED-23G was 128 μg/ml; for strain Shjt42b, it was 256 μg/ml; and for strain ED-23G, it reached 512 μg/ml. These results suggest that CMCh-Ag^⁺^, but not its individual components, possesses significant activity in reducing *A. fumigatus* biofilm biomass or impairing biofilm-associated viability under these experimental conditions. To further investigate the antimicrobial mechanism of agents in resistant *A. fumigatus*, we conducted experiments to measure reactive oxygen species (ROS). The results presented in Fig. 3C indicate that, compared with the control group, treatment with CMCh alone had a negligible effect on the levels of ROS within the fungal cells of drug-resistant *A. fumigatus* and may even lead to a slight decrease. In contrast, treatment with Ag^+^ significantly promoted the production of ROS in drug-resistant *A. fumigatus*. Notably, when CMCh and Ag^⁺^ were combined, there was a remarkable increase in ROS levels within the fungi—almost doubling. The observed increase in ROS fluorescence intensity upon CMCh-Ag^⁺^ treatment suggests an association between ROS accumulation and the antifungal activity observed in our assays. This elevated ROS level may reflect a significant oxidative stress response induced by the composite in drug-resistant *A. fumigatus*, which correlates with the observed fungal growth inhibition and cell death.

## Discussion

In recent years, the extensive use of antifungal agents has led to an increasingly prominent problem of drug resistance in *A. fumigatus* against conventional therapies. This crisis of resistance arises from various molecular adaptations, including target-site mutations (notably alterations in cyp51A that confer azole resistance) (Pfaller et al., 2023; Pham et al., 2014), overexpression of efflux pumps, and biofilm-mediated tolerance facilitated by fortification of the extracellular matrix (Moazam et al., 2020). This alarming statistic underscores the urgent need for novel antifungal agents with distinct pharmacophores and a reduced propensity for developing resistance (Fisher et al., 2022; Lestrade et al., 2019). Based on a systematic analysis of existing literature and prior research on chitosan-silver composites, we have deliberately extended the antimicrobial application of our composite to target the pathogenic fungus *A. fumigatus*, including its drug-resistant strains. This design follows an effective strategy of combining biopolymers with inorganic antimicrobial agents to achieve synergistic effects and sustained release. The synthesized CMCh-Ag^⁺^ composite not only retains the inherent biocompatibility and biodegradability of the chitosan matrix (Chen et al., 2024a; Li et al., 2023) but also fully incorporates the broad spectrum antibacterial activity, high efficacy, and sustained release properties of silver ions, as previously demonstrated by other researchers (Durán et al., 2016; Rai et al., 2009; Sotiriou and Pratsinis, 2010). As a result, genuine synergy and enhanced performance are achieved at both structural and functional levels.

The characterization experiments indicate that CMCh-Ag^⁺^ exhibits a unique particle size distribution, ultraviolet and infrared absorption characteristics, as well as distinct micro-morphology. It also demonstrates excellent dispersibility, which facilitates broader contact with microorganisms and enhances antibacterial efficacy. The spectral peak shifts further confirm the interaction between carboxymethyl chitosan and silver ions. These findings are in agreement with prior research (Wahid et al., 2017). Moreover, In vitro antibacterial tests including MIC, MFC, and cell growth curves, along with In vivo antibacterial experiments using *G. mellonella*, demonstrate that CMCh, Ag^⁺^, and CMCh-Ag^⁺^ all possess antibacterial effects against *A. fumigatus*; among these, CMCh-Ag^⁺^ shows the most pronounced antifungal activity.

In terms of antifungal mechanism-related activity, this study provides new observations: the activity of CMCh-Ag^⁺^ is closely associated with its ability to reduce fungal biofilm biomass and modulate reactive oxygen species (ROS) levels. Biofilms are a critical factor contributing to fungal drug resistance and persistent infections (Nett and Pohl, 2021). Although previous studies have reported the inhibitory effects of silver nanoparticles (El Dougdoug et al., 2025) or chitosan (Kvasničková et al., 2016) on bacterial biofilms, evidence regarding their efficacy against fungal biofilms—particularly those of *A. fumigatus*—remains limited. This study confirms previous findings that neither CMCh nor Ag^⁺^ alone caused a significant reduction in *A. fumigatus* biofilm biomass in our crystal violet assay, whereas the CMCh-Ag^⁺^ composite significantly decreased the staining intensity in a concentration-dependent manner. These results suggest its potential advantage in reducing biofilm biomass, consistent with prior reports on similar composite systems (Shehabeldine et al., 2022).

Regarding mechanism-related observations, Ag^+^ induced ROS generation is commonly reported as a phenomenon associated with antimicrobial activity (Dakal et al., 2016). In line with this, our study observed that CMCh-Ag^+^ treatment led to a more pronounced increase in intracellular ROS fluorescence compared to Ag^+^ alone. This suggests that ROS accumulation is a prominent feature of the cellular response to CMCh-Ag^+^ stress. While oxidative stress is a potential contributor to antimicrobial outcomes, the current data establish an association rather than a definitive causative role for ROS in the fungal killing mediated by CMCh-Ag^+^. This association between ROS accumulation and reduced biofilm biomass warrants further investigation to elucidate potential causal relationships and detailed mechanisms.

The *G. mellonella* possesses a highly developed immune system that shares similarities with the mammalian innate immune system. This immunological parallelism implies that pathogens capable of infecting and killing *G. mellonella*, often rely on similar virulence factors to evade host immune defenses (Curtis et al., 2022). Consequently, evaluating pathogen virulence and assessing the therapeutic efficacy of agents in the *G. mellonella* model holds high biological relevance. Compared to mammalian models, the *G. mellonella* model offers significantly lower rearing costs. Their relatively large size (approximately 2–3 cm in length) facilitates precise microinjection for pathogen inoculation and agent administration (Tsai et al., 2016). Therefore, the data obtained using this model provide preliminary In vivo evidence supporting the efficacy and biocompatibility of the CMCh-Ag^+^ nanocomposite, which in turn strengthens the rationale for and informs the design of subsequent, more complex mammalian model studies.

Despite the encouraging results, this study has several limitations. First, although the *G. mellonella* model provided valuable insights, it must be complemented by mammalian IA models, including a neutropenic murine inhalation model. These are necessary to quantify lung fungal burden, cytokine profiles, and histopathology at clinically relevant doses (Darlow et al., 2025). Second, we acknowledge that the interpretation of particle size measurement data requires further validation. The observed particle sizes in the silver-containing solution should ideally be cross-validated using higher-resolution imaging techniques to confirm their morphology and aggregation state, as well as to precisely characterize the chemical equilibrium within the solution. The data presented in this study indicate the size distribution of molecular assemblies in solution but do not independently verify colloid formation. Third, a comprehensive safety profile is required, which should include assessments of hemocompatibility, cytotoxicity toward pulmonary epithelial and immune cells, the immunogenicity of CMCh, and the evaluation of silver accumulation and excretion. Finally, combination therapy studies with triazoles or echinocandins should be conducted to evaluate potential synergistic effects (Denardi et al., 2017).

In summary, this study successfully developed a novel silver ion composite material, CMCh-Ag^⁺^, which exhibits excellent anti *A. fumigatus* performance. The composite demonstrates high antimicrobial activity both in vitro and in vivo, and its antifungal activity is associated with reduced biofilm biomass and elevated intracellular ROS levels. These findings provide an important theoretical basis and preclinical rationale for developing new strategies against drug resistant fungal infections. Future studies employing mammalian models of invasive pulmonary aspergillosis will be essential to evaluate the pharmacokinetic profiles, lung-specific biodistribution, and comprehensive safety of CMCh-Ag^⁺^ before any consideration of its potential clinical translation as an inhalation therapy.

## Figures and Tables

**Table 1.** Drug susceptibility test of CMCh, Ag^⁺^, and CMCh-Ag^⁺^

Strains	MIC (μg/ml)			MFC (μg/ml)		
	CMCh	Ag^+^	CMCh-Ag^+^	CMCh	Ag^+^	CMCh-Ag^+^
A1161	4096	64	16	＞ 512	＞ 512	128
Shjt40	2048	64	32	＞ 512	＞ 512	256
Shjt42b	8192	128	32	＞ 512	＞ 512	256
ED-22G	8192	64	64	＞ 512	＞ 512	512
ED-23G	8192	128	32	＞ 512	＞ 512	256
